# *cis*-Bis[*N*-(2-furoyl)-*N*′,*N*′-diphenyl­thio­ureato-κ^2^ *O*,*S*]nickel(II)

**DOI:** 10.1107/S160053680900302X

**Published:** 2009-01-31

**Authors:** Hiram Pérez, Rodrigo S. Corrêa, Ana María Plutín, Osmar Calderón, Julio Duque

**Affiliations:** a Departamento de Química Inorgánica, Facultad de Química, Universidad de la Habana, Habana 10400, Cuba; b Grupo de Cristalografía, Instituto de Física de São Carlos, Universidade de São Paulo, São Carlos, Brazil; c Laboratorio de Síntesis Orgánica, Facultad de Química, Universidad de la Habana, Habana 10400, Cuba; d Instituto de Ciencia y Tecnología de Materiales, Universidad de la Habana, Habana 10400, Cuba

## Abstract

In the title compound, [Ni(C_18_H_13_N_2_O_2_S)_2_], the Ni^II^ atom is coordinated by the S and O atoms of two *N*-furoyl-*N*′,*N*′-diphenyl­thio­ureate ligands in a slightly distorted square-planar coordination geometry. The two O and two S atoms are *cis* to each other.

## Related literature

For general background, see: Arslan *et al.* (2006 ▶). For related structures, see: Jia *et al.* (2007 ▶); Pérez *et al.* (2008 ▶). For the synthesis of the ligand, see: Hernández *et al.* (2003 ▶).
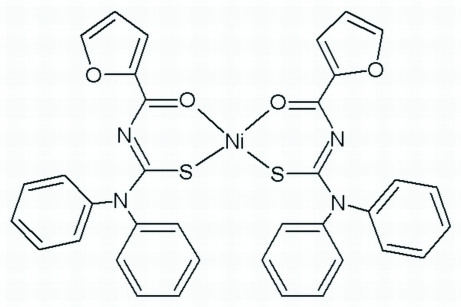


## Experimental

### 

#### Crystal data

[Ni(C_18_H_13_N_2_O_2_S)_2_]
*M* *_r_* = 701.46
Triclinic, 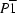

*a* = 10.0458 (2) Å
*b* = 11.0030 (3) Å
*c* = 15.9718 (3) Å
α = 72.755 (2)°
β = 88.792 (2)°
γ = 74.874 (1)°
*V* = 1624.61 (7) Å^3^
*Z* = 2
Mo *K*α radiation
μ = 0.77 mm^−1^
*T* = 294 K
0.15 × 0.09 × 0.06 mm

#### Data collection

Nonius KappaCCD diffractometer
Absorption correction: Gaussian (Coppens *et al.*, 1965 ▶) *T*_min_ = 0.955, *T*_max_ = 0.980
12907 measured reflections
7027 independent reflections
5083 reflections with *I* > 2σ(*I*)
*R*_int_ = 0.053

#### Refinement

*R*[*F* ^2^ > 2σ(*F* ^2^)] = 0.064
*wR*(*F* ^2^) = 0.145
*S* = 1.22
7027 reflections
424 parameters
H-atom parameters constrained
Δρ_max_ = 0.33 e Å^−3^
Δρ_min_ = −0.46 e Å^−3^

### 

Data collection: *COLLECT* (Enraf–Nonius, 2000 ▶); cell refinement: *DENZO*/*SCALEPACK* (Otwinowski & Minor, 1997 ▶); data reduction: *DENZO*/*SCALEPACK*; program(s) used to solve structure: *SHELXS97* (Sheldrick, 2008 ▶); program(s) used to refine structure: *SHELXL97* (Sheldrick, 2008 ▶); molecular graphics: *ORTEP-3* (Farrugia, 1997 ▶); software used to prepare material for publication: *WinGX* (Farrugia, 1999 ▶).

## Supplementary Material

Crystal structure: contains datablocks global, I. DOI: 10.1107/S160053680900302X/gk2187sup1.cif

Structure factors: contains datablocks I. DOI: 10.1107/S160053680900302X/gk2187Isup2.hkl

Additional supplementary materials:  crystallographic information; 3D view; checkCIF report

## Figures and Tables

**Table:** 

Ni1—O3	1.870 (2)
Ni1—O1	1.872 (2)
Ni1—S1	2.1412 (9)
Ni1—S2	2.1452 (9)

**Table:** 

O3—Ni1—O1	84.21 (9)
O3—Ni1—S1	176.26 (8)
O1—Ni1—S1	95.90 (7)
O3—Ni1—S2	95.82 (7)
O1—Ni1—S2	176.87 (8)
S1—Ni1—S2	84.28 (3)

**Fig. 1.**
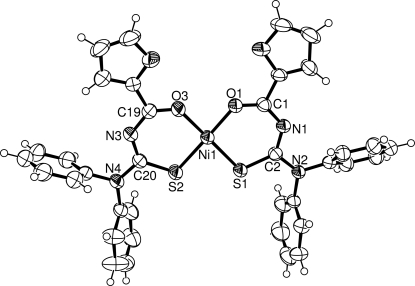
The molecular structure of the title compound. Displacement ellipsoids are drawn at the 50% probability level.

**Fig. 2.**
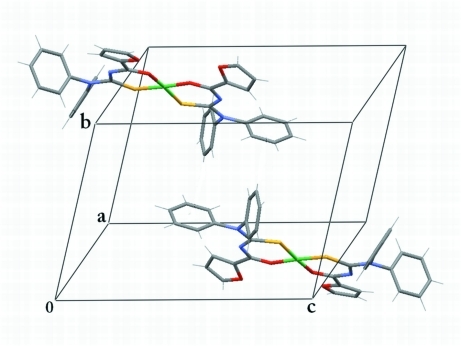
View of the unit cell of the title complex.

## supplementary crystallographic information

### Comment

*N*-Acyl-*N*',*N*'-disubstituted thioureas are well known as chelating agents. Over recent years, many transition metal complexes with *N*-benzoyl- and *N*-furoyl-*N*',*N*'-disubstituted thioureas have been reported (Jia *et al.*, 2007). During the complex formation, the ligand is deprotonated, which results in a neutral complex with a six-membered ring chelating metal ion. In this paper, we report the crystal structure of the title compound.

In the structure of complex, the two furoylthiourea ligands have *cis* arrangement when bonded to the central Ni^II^ ion as shown in Fig. 1. The complex coordination geometry is a slightly distorted square-planar as reflected by the angles O3—Ni1—S1 [176.26 (8)°] and O1—Ni1—S2 [176.87 (8)°].

Selected geometric parameters are listed in Table 1. The Ni—S and Ni—O bond lengths lie within the range of those found in the related structures (Pérez *et al.*, 2008). The lengths of C—O, C—S and C—N bonds in the chelate ring are between characteristic single and double bond lengths (Arslan *et al.*, 2006), which are shorter than single and longer than double bonds. Fig. 2 shows the arrangement of the complex molecules in the unit cell.

### Experimental

*N*-Furoyl-*N*',*N*'-diphenylthiourea ligand was synthesized according to a procedure described by Hernández *et al.* (2003), by converting furoyl chloride into furoyl isothiocyanate and then condensing with an appropriate amine. To an ethanol solution (30 ml) containing the ligand (0.64 g, 2 mmol) was added an ethanol solution of Ni(CH_3_COO)_2_.4H_2_O (0.25 g, 1 mmol). The solution was stirred at room temperature for 2 h, and at once a solution of NaOH (1 N) was added to adjust pH to the neutral value. The mixture was filtered and the filtrate was evaporated under reduced pressure to give a red solid, which was washed with acetone. Single crystals were obtained by slow evaporation of a chloroform/*N*,*N*-diphenylformamide solution (1:1, *v*/*v*) of the complex.

### Refinement

H atoms were positioned geometrically and refined as riding atoms, with C—H = 0.93 Å and U_iso_(H) = 1.2U_eq_(C).

### Crystal data

**Table:**

[Ni(C_18_H_13_N_2_O_2_S)_2_]	*Z* = 2
*M**_r_* = 701.46	*F*(000) = 724
Triclinic, *P*1	*D*_x_ = 1.434 Mg m^−^^3^
Hall symbol: -P 1	Mo *K*α radiation, λ = 0.71073 Å
*a* = 10.0458 (2) Å	Cell parameters from 83255 reflections
*b* = 11.0030 (3) Å	θ = 2.9–27.1°
*c* = 15.9718 (3) Å	µ = 0.77 mm^−^^1^
α = 72.755 (2)°	*T* = 294 K
β = 88.792 (2)°	Prism, red
γ = 74.874 (1)°	0.14 × 0.09 × 0.06 mm
*V* = 1624.61 (7) Å^3^	

### Data collection

**Table:**

Nonius KappaCCD diffractometer	5083 reflections with *I* > 2σ(*I*)
φ and ω scans	*R*_int_ = 0.053
Absorption correction: gaussian (Coppens *et al.*, 1965)	θ_max_ = 27.1°, θ_min_ = 3.1°
*T*_min_ = 0.955, *T*_max_ = 0.980	*h* = −12→12
12907 measured reflections	*k* = −13→14
7027 independent reflections	*l* = −17→20

### Refinement

**Table:**

Refinement on *F*^2^	0 restraints
Least-squares matrix: full	H-atom parameters constrained
*R*[*F*^2^ > 2σ(*F*^2^)] = 0.064	*w* = 1/[σ^2^(*F*_o_^2^) + (0.0511*P*)^2^ + 0.2693*P*] where *P* = (*F*_o_^2^ + 2*F*_c_^2^)/3
*wR*(*F*^2^) = 0.145	(Δ/σ)_max_ < 0.001
*S* = 1.22	Δρ_max_ = 0.33 e Å^−^^3^
7027 reflections	Δρ_min_ = −0.45 e Å^−^^3^
424 parameters	

### Special details

**Table:**

Geometry. All e.s.d.'s (except the e.s.d. in the dihedral angle between two l.s. planes) are estimated using the full covariance matrix. The cell e.s.d.'s are taken into account individually in the estimation of e.s.d.'s in distances, angles and torsion angles; correlations between e.s.d.'s in cell parameters are only used when they are defined by crystal symmetry. An approximate (isotropic) treatment of cell e.s.d.'s is used for estimating e.s.d.'s involving l.s. planes.

### Fractional atomic coordinates and isotropic or equivalent isotropic displacement parameters (Å^2^)

**Table:**

	*x*	*y*	*z*	*U*_iso_*/*U*_eq_	
Ni1	0.10992 (4)	0.16148 (4)	0.22008 (2)	0.04396 (16)	
S2	0.17245 (9)	0.12133 (9)	0.09958 (6)	0.0542 (3)	
S1	0.27714 (9)	−0.00834 (9)	0.27898 (6)	0.0562 (3)	
C25	0.1818 (3)	0.1120 (3)	−0.0847 (2)	0.0468 (8)	
C3	0.0498 (3)	0.2104 (3)	0.4681 (2)	0.0461 (8)	
O2	−0.0374 (2)	0.3346 (2)	0.43990 (16)	0.0594 (6)	
C5	−0.0320 (4)	0.2626 (5)	0.5856 (3)	0.0802 (13)	
H5	−0.0504	0.2582	0.6436	0.096*	
O1	0.0615 (2)	0.2029 (2)	0.32411 (14)	0.0539 (6)	
C16	0.4337 (4)	−0.2623 (4)	0.7121 (2)	0.0608 (10)	
H16	0.4447	−0.2931	0.7731	0.073*	
C9	0.5753 (5)	−0.4263 (4)	0.3721 (3)	0.0758 (12)	
H9	0.568	−0.5109	0.3778	0.091*	
O3	−0.0437 (2)	0.3025 (2)	0.16983 (14)	0.0551 (6)	
N4	0.0672 (3)	0.2042 (3)	−0.06320 (16)	0.0485 (7)	
O4	−0.2840 (2)	0.4849 (2)	0.14960 (16)	0.0649 (7)	
C15	0.5424 (4)	−0.2347 (4)	0.6641 (2)	0.0592 (9)	
H15	0.6265	−0.2459	0.6926	0.071*	
N2	0.3911 (3)	−0.1353 (3)	0.43740 (16)	0.0433 (6)	
N1	0.2152 (3)	0.0424 (3)	0.43601 (16)	0.0427 (6)	
C31	−0.0362 (3)	0.2842 (3)	−0.1339 (2)	0.0459 (8)	
C17	0.3089 (4)	−0.2447 (4)	0.6709 (2)	0.0593 (10)	
H17	0.2351	−0.2628	0.7039	0.071*	
C22	−0.3081 (4)	0.5271 (4)	0.0059 (3)	0.0696 (11)	
H22	−0.2911	0.5258	−0.0513	0.084*	
C21	−0.2258 (3)	0.4548 (3)	0.0779 (2)	0.0467 (8)	
C13	0.4024 (3)	−0.1739 (3)	0.5320 (2)	0.0420 (7)	
C7	0.4894 (3)	−0.2189 (3)	0.3976 (2)	0.0456 (8)	
C12	0.5971 (4)	−0.1767 (4)	0.3554 (2)	0.0641 (10)	
H12	0.6045	−0.0921	0.3495	0.077*	
C18	0.2929 (3)	−0.2002 (3)	0.5805 (2)	0.0491 (8)	
H18	0.2082	−0.188	0.5524	0.059*	
N3	−0.0573 (3)	0.3195 (3)	0.02013 (16)	0.0476 (7)	
C1	0.1123 (3)	0.1489 (3)	0.4017 (2)	0.0424 (7)	
C26	0.3131 (4)	0.1250 (4)	−0.0810 (2)	0.0650 (10)	
H26	0.3293	0.1915	−0.0611	0.078*	
C36	−0.1497 (4)	0.2426 (4)	−0.1455 (2)	0.0574 (9)	
H36	−0.1617	0.1644	−0.1075	0.069*	
C14	0.5278 (3)	−0.1902 (3)	0.5730 (2)	0.0521 (9)	
H14	0.6016	−0.1716	0.5402	0.063*	
C20	0.0522 (3)	0.2238 (3)	0.0169 (2)	0.0439 (8)	
C4	0.0562 (4)	0.1639 (4)	0.5560 (2)	0.0667 (11)	
H4	0.1089	0.082	0.5907	0.08*	
C19	−0.0975 (3)	0.3501 (3)	0.0924 (2)	0.0463 (8)	
C32	−0.0178 (4)	0.3994 (4)	−0.1894 (2)	0.0598 (10)	
H32	0.0595	0.4273	−0.1816	0.072*	
C27	0.4219 (4)	0.0402 (5)	−0.1067 (3)	0.0723 (12)	
H27	0.5106	0.0504	−0.1044	0.087*	
C35	−0.2467 (4)	0.3177 (4)	−0.2142 (3)	0.0692 (11)	
H35	−0.3235	0.2895	−0.2229	0.083*	
C23	−0.4244 (4)	0.6049 (4)	0.0329 (3)	0.0777 (13)	
H23	−0.4994	0.6652	−0.0028	0.093*	
C8	0.4766 (4)	−0.3430 (4)	0.4059 (2)	0.0582 (9)	
H8	0.4028	−0.3707	0.4338	0.07*	
C11	0.6944 (4)	−0.2612 (5)	0.3217 (3)	0.0796 (13)	
H11	0.7676	−0.2335	0.2929	0.095*	
C30	0.1591 (4)	0.0128 (4)	−0.1135 (3)	0.0716 (12)	
H30	0.0705	0.0024	−0.116	0.086*	
C10	0.6831 (5)	−0.3862 (5)	0.3306 (3)	0.0824 (15)	
H10	0.749	−0.4433	0.3083	0.099*	
C2	0.2900 (3)	−0.0298 (3)	0.3898 (2)	0.0406 (7)	
C33	−0.1163 (5)	0.4739 (4)	−0.2575 (2)	0.0729 (12)	
H33	−0.1051	0.5524	−0.2957	0.087*	
C6	−0.0839 (4)	0.3632 (4)	0.5149 (3)	0.0714 (12)	
H6	−0.1441	0.4428	0.516	0.086*	
C28	0.4000 (5)	−0.0574 (4)	−0.1351 (3)	0.0759 (12)	
H28	0.4735	−0.1146	−0.152	0.091*	
C24	−0.4066 (4)	0.5758 (4)	0.1190 (3)	0.0731 (12)	
H24	−0.4696	0.6127	0.1541	0.088*	
C34	−0.2292 (5)	0.4322 (4)	−0.2686 (3)	0.0727 (12)	
H34	−0.2951	0.4831	−0.3141	0.087*	
C29	0.2695 (5)	−0.0716 (4)	−0.1389 (3)	0.0892 (15)	
H29	0.2545	−0.1387	−0.1587	0.107*	

### Atomic displacement parameters (Å^2^)

**Table:**

	*U*^11^	*U*^22^	*U*^33^	*U*^12^	*U*^13^	*U*^23^
Ni1	0.0427 (3)	0.0473 (3)	0.0365 (3)	−0.00044 (19)	0.00223 (18)	−0.01466 (19)
S2	0.0514 (5)	0.0615 (6)	0.0397 (5)	0.0050 (4)	0.0030 (4)	−0.0179 (4)
S1	0.0577 (6)	0.0587 (6)	0.0390 (5)	0.0128 (4)	−0.0016 (4)	−0.0198 (4)
C25	0.0448 (19)	0.054 (2)	0.0376 (17)	−0.0041 (16)	0.0069 (14)	−0.0156 (15)
C3	0.0386 (18)	0.050 (2)	0.050 (2)	0.0001 (16)	0.0030 (15)	−0.0261 (16)
O2	0.0571 (15)	0.0555 (15)	0.0630 (16)	0.0006 (12)	0.0088 (12)	−0.0282 (12)
C5	0.074 (3)	0.107 (4)	0.062 (3)	0.003 (3)	0.009 (2)	−0.053 (3)
O1	0.0560 (14)	0.0540 (14)	0.0398 (13)	0.0092 (11)	−0.0010 (11)	−0.0170 (11)
C16	0.085 (3)	0.051 (2)	0.0396 (19)	−0.005 (2)	−0.001 (2)	−0.0148 (16)
C9	0.092 (3)	0.055 (3)	0.072 (3)	0.012 (2)	−0.007 (2)	−0.033 (2)
O3	0.0536 (14)	0.0612 (15)	0.0385 (13)	0.0073 (12)	−0.0005 (11)	−0.0164 (11)
N4	0.0485 (16)	0.0541 (17)	0.0400 (15)	−0.0026 (14)	0.0014 (12)	−0.0197 (13)
O4	0.0590 (16)	0.0630 (17)	0.0567 (15)	0.0087 (13)	0.0086 (12)	−0.0164 (13)
C15	0.063 (2)	0.059 (2)	0.054 (2)	−0.0102 (19)	−0.0128 (19)	−0.0181 (18)
N2	0.0391 (14)	0.0483 (16)	0.0365 (14)	0.0020 (12)	0.0014 (11)	−0.0161 (12)
N1	0.0402 (15)	0.0449 (16)	0.0402 (15)	−0.0032 (13)	0.0032 (12)	−0.0157 (12)
C31	0.0480 (19)	0.048 (2)	0.0372 (17)	−0.0029 (16)	0.0041 (15)	−0.0148 (15)
C17	0.063 (2)	0.058 (2)	0.048 (2)	−0.0072 (19)	0.0146 (18)	−0.0113 (17)
C22	0.068 (3)	0.064 (3)	0.065 (3)	0.009 (2)	−0.016 (2)	−0.025 (2)
C21	0.0481 (19)	0.0432 (19)	0.0446 (19)	−0.0021 (16)	0.0011 (15)	−0.0156 (15)
C13	0.0431 (18)	0.0412 (18)	0.0402 (17)	−0.0040 (15)	0.0019 (14)	−0.0164 (14)
C7	0.0408 (18)	0.050 (2)	0.0373 (17)	0.0059 (15)	0.0032 (14)	−0.0159 (15)
C12	0.052 (2)	0.076 (3)	0.060 (2)	−0.005 (2)	0.0154 (18)	−0.026 (2)
C18	0.0429 (19)	0.056 (2)	0.0444 (19)	−0.0071 (16)	0.0036 (15)	−0.0140 (16)
N3	0.0529 (17)	0.0500 (17)	0.0378 (15)	−0.0060 (14)	0.0036 (13)	−0.0170 (13)
C1	0.0399 (18)	0.0474 (19)	0.0410 (18)	−0.0105 (16)	0.0067 (14)	−0.0161 (15)
C26	0.059 (2)	0.086 (3)	0.060 (2)	−0.020 (2)	0.0100 (19)	−0.036 (2)
C36	0.057 (2)	0.056 (2)	0.056 (2)	−0.0100 (19)	−0.0011 (18)	−0.0170 (18)
C14	0.049 (2)	0.056 (2)	0.050 (2)	−0.0113 (17)	−0.0010 (16)	−0.0159 (17)
C20	0.0463 (19)	0.048 (2)	0.0381 (17)	−0.0114 (16)	0.0077 (14)	−0.0151 (15)
C4	0.062 (2)	0.085 (3)	0.046 (2)	0.007 (2)	−0.0003 (18)	−0.030 (2)
C19	0.0476 (19)	0.0430 (19)	0.0439 (19)	−0.0068 (16)	0.0017 (15)	−0.0110 (15)
C32	0.066 (2)	0.056 (2)	0.057 (2)	−0.015 (2)	0.0057 (19)	−0.0177 (19)
C27	0.049 (2)	0.104 (4)	0.067 (3)	−0.013 (2)	0.0125 (19)	−0.037 (3)
C35	0.055 (2)	0.075 (3)	0.078 (3)	−0.003 (2)	−0.010 (2)	−0.034 (2)
C23	0.061 (3)	0.059 (3)	0.097 (4)	0.016 (2)	−0.026 (2)	−0.025 (2)
C8	0.065 (2)	0.054 (2)	0.053 (2)	−0.0037 (19)	0.0029 (18)	−0.0217 (18)
C11	0.054 (2)	0.107 (4)	0.068 (3)	0.000 (3)	0.017 (2)	−0.031 (3)
C30	0.063 (3)	0.069 (3)	0.100 (3)	−0.022 (2)	0.023 (2)	−0.047 (2)
C10	0.064 (3)	0.108 (4)	0.054 (3)	0.024 (3)	0.004 (2)	−0.035 (3)
C2	0.0364 (17)	0.0440 (19)	0.0410 (17)	−0.0074 (15)	0.0015 (14)	−0.0149 (15)
C33	0.095 (3)	0.053 (2)	0.052 (2)	−0.003 (2)	0.004 (2)	−0.0043 (19)
C6	0.061 (2)	0.077 (3)	0.083 (3)	0.000 (2)	0.012 (2)	−0.052 (3)
C28	0.070 (3)	0.073 (3)	0.076 (3)	0.000 (2)	0.022 (2)	−0.027 (2)
C24	0.057 (3)	0.059 (3)	0.090 (3)	0.010 (2)	0.007 (2)	−0.025 (2)
C34	0.074 (3)	0.070 (3)	0.060 (3)	0.012 (2)	−0.016 (2)	−0.026 (2)
C29	0.084 (3)	0.068 (3)	0.135 (4)	−0.021 (3)	0.037 (3)	−0.061 (3)

### Geometric parameters (Å, °)

**Table:**

Ni1—O3	1.870 (2)	C22—C23	1.400 (5)
Ni1—O1	1.872 (2)	C22—H22	0.93
Ni1—S1	2.1412 (9)	C21—C19	1.456 (4)
Ni1—S2	2.1452 (9)	C13—C18	1.376 (4)
S2—C20	1.717 (3)	C13—C14	1.376 (4)
S1—C2	1.718 (3)	C7—C8	1.372 (5)
C25—C26	1.368 (5)	C7—C12	1.374 (5)
C25—C30	1.373 (5)	C12—C11	1.383 (5)
C25—N4	1.433 (4)	C12—H12	0.93
C3—C4	1.341 (5)	C18—H18	0.93
C3—O2	1.366 (4)	N3—C20	1.322 (4)
C3—C1	1.468 (4)	N3—C19	1.323 (4)
O2—C6	1.370 (4)	C26—C27	1.381 (5)
C5—C6	1.325 (6)	C26—H26	0.93
C5—C4	1.405 (5)	C36—C35	1.388 (5)
C5—H5	0.93	C36—H36	0.93
O1—C1	1.257 (4)	C14—H14	0.93
C16—C15	1.368 (5)	C4—H4	0.93
C16—C17	1.369 (5)	C32—C33	1.388 (5)
C16—H16	0.93	C32—H32	0.93
C9—C10	1.361 (6)	C27—C28	1.350 (6)
C9—C8	1.381 (5)	C27—H27	0.93
C9—H9	0.93	C35—C34	1.354 (6)
O3—C19	1.261 (4)	C35—H35	0.93
N4—C20	1.357 (4)	C23—C24	1.321 (6)
N4—C31	1.452 (4)	C23—H23	0.93
O4—C24	1.360 (4)	C8—H8	0.93
O4—C21	1.364 (4)	C11—C10	1.374 (6)
C15—C14	1.387 (5)	C11—H11	0.93
C15—H15	0.93	C30—C29	1.387 (5)
N2—C2	1.360 (4)	C30—H30	0.93
N2—C7	1.440 (4)	C10—H10	0.93
N2—C13	1.441 (4)	C33—C34	1.363 (6)
N1—C2	1.323 (4)	C33—H33	0.93
N1—C1	1.324 (4)	C6—H6	0.93
C31—C36	1.369 (5)	C28—C29	1.365 (6)
C31—C32	1.370 (5)	C28—H28	0.93
C17—C18	1.379 (4)	C24—H24	0.93
C17—H17	0.93	C34—H34	0.93
C22—C21	1.339 (5)	C29—H29	0.93
			
O3—Ni1—O1	84.21 (9)	N1—C1—C3	112.0 (3)
O3—Ni1—S1	176.26 (8)	C25—C26—C27	120.5 (4)
O1—Ni1—S1	95.90 (7)	C25—C26—H26	119.7
O3—Ni1—S2	95.82 (7)	C27—C26—H26	119.7
O1—Ni1—S2	176.87 (8)	C31—C36—C35	119.7 (4)
S1—Ni1—S2	84.28 (3)	C31—C36—H36	120.2
C20—S2—Ni1	108.55 (11)	C35—C36—H36	120.2
C2—S1—Ni1	108.64 (11)	C13—C14—C15	119.0 (3)
C26—C25—C30	119.3 (3)	C13—C14—H14	120.5
C26—C25—N4	120.9 (3)	C15—C14—H14	120.5
C30—C25—N4	119.7 (3)	N3—C20—N4	114.0 (3)
C4—C3—O2	110.4 (3)	N3—C20—S2	128.9 (2)
C4—C3—C1	131.4 (3)	N4—C20—S2	117.1 (2)
O2—C3—C1	118.1 (3)	C3—C4—C5	106.7 (4)
C3—O2—C6	105.1 (3)	C3—C4—H4	126.6
C6—C5—C4	106.7 (4)	C5—C4—H4	126.6
C6—C5—H5	126.7	O3—C19—N3	130.4 (3)
C4—C5—H5	126.7	O3—C19—C21	116.7 (3)
C1—O1—Ni1	131.4 (2)	N3—C19—C21	112.8 (3)
C15—C16—C17	120.4 (3)	C31—C32—C33	119.1 (4)
C15—C16—H16	119.8	C31—C32—H32	120.4
C17—C16—H16	119.8	C33—C32—H32	120.4
C10—C9—C8	120.9 (4)	C28—C27—C26	120.3 (4)
C10—C9—H9	119.6	C28—C27—H27	119.8
C8—C9—H9	119.6	C26—C27—H27	119.8
C19—O3—Ni1	131.6 (2)	C34—C35—C36	119.7 (4)
C20—N4—C25	124.4 (3)	C34—C35—H35	120.1
C20—N4—C31	118.9 (3)	C36—C35—H35	120.1
C25—N4—C31	116.6 (2)	C24—C23—C22	106.4 (4)
C24—O4—C21	105.7 (3)	C24—C23—H23	126.8
C16—C15—C14	120.3 (3)	C22—C23—H23	126.8
C16—C15—H15	119.8	C7—C8—C9	118.9 (4)
C14—C15—H15	119.8	C7—C8—H8	120.5
C2—N2—C7	122.7 (2)	C9—C8—H8	120.5
C2—N2—C13	121.6 (2)	C10—C11—C12	120.1 (4)
C7—N2—C13	115.6 (2)	C10—C11—H11	120
C2—N1—C1	123.9 (3)	C12—C11—H11	120
C36—C31—C32	120.5 (3)	C25—C30—C29	119.4 (4)
C36—C31—N4	119.8 (3)	C25—C30—H30	120.3
C32—C31—N4	119.7 (3)	C29—C30—H30	120.3
C16—C17—C18	119.9 (3)	C9—C10—C11	119.9 (4)
C16—C17—H17	120.1	C9—C10—H10	120
C18—C17—H17	120.1	C11—C10—H10	120
C21—C22—C23	107.3 (4)	N1—C2—N2	114.9 (3)
C21—C22—H22	126.4	N1—C2—S1	129.1 (2)
C23—C22—H22	126.4	N2—C2—S1	116.0 (2)
C22—C21—O4	109.6 (3)	C34—C33—C32	120.1 (4)
C22—C21—C19	132.7 (3)	C34—C33—H33	120
O4—C21—C19	117.7 (3)	C32—C33—H33	120
C18—C13—C14	120.5 (3)	C5—C6—O2	111.1 (3)
C18—C13—N2	121.0 (3)	C5—C6—H6	124.5
C14—C13—N2	118.4 (3)	O2—C6—H6	124.5
C8—C7—C12	120.9 (3)	C27—C28—C29	119.7 (4)
C8—C7—N2	118.7 (3)	C27—C28—H28	120.1
C12—C7—N2	120.3 (3)	C29—C28—H28	120.1
C7—C12—C11	119.3 (4)	C23—C24—O4	111.1 (4)
C7—C12—H12	120.3	C23—C24—H24	124.5
C11—C12—H12	120.3	O4—C24—H24	124.5
C13—C18—C17	119.9 (3)	C35—C34—C33	120.9 (4)
C13—C18—H18	120.1	C35—C34—H34	119.6
C17—C18—H18	120.1	C33—C34—H34	119.6
C20—N3—C19	124.5 (3)	C28—C29—C30	120.7 (4)
O1—C1—N1	131.0 (3)	C28—C29—H29	119.7
O1—C1—C3	117.0 (3)	C30—C29—H29	119.7
			
C4—C3—O2—C6	0.5 (4)	C25—N4—C20—S2	−5.4 (4)
C1—C3—O2—C6	177.2 (3)	C31—N4—C20—S2	176.9 (2)
C26—C25—N4—C20	−62.4 (5)	Ni1—S2—C20—N3	4.5 (3)
C30—C25—N4—C20	120.9 (4)	Ni1—S2—C20—N4	−173.9 (2)
C26—C25—N4—C31	115.4 (4)	O2—C3—C4—C5	0.4 (5)
C30—C25—N4—C31	−61.4 (4)	C1—C3—C4—C5	−175.7 (3)
C17—C16—C15—C14	−0.8 (6)	C6—C5—C4—C3	−1.3 (5)
C20—N4—C31—C36	−89.2 (4)	Ni1—O3—C19—N3	−4.2 (6)
C25—N4—C31—C36	93.0 (4)	Ni1—O3—C19—C21	174.8 (2)
C20—N4—C31—C32	92.1 (4)	C20—N3—C19—O3	2.3 (6)
C25—N4—C31—C32	−85.8 (4)	C20—N3—C19—C21	−176.7 (3)
C15—C16—C17—C18	0.6 (6)	C22—C21—C19—O3	179.0 (4)
C23—C22—C21—O4	−0.9 (5)	O4—C21—C19—O3	−5.6 (5)
C23—C22—C21—C19	174.7 (4)	C22—C21—C19—N3	−1.8 (6)
C24—O4—C21—C22	1.5 (4)	O4—C21—C19—N3	173.5 (3)
C24—O4—C21—C19	−174.8 (3)	C36—C31—C32—C33	0.3 (5)
C2—N2—C13—C18	55.4 (4)	N4—C31—C32—C33	179.0 (3)
C7—N2—C13—C18	−122.0 (3)	C25—C26—C27—C28	−0.5 (6)
C2—N2—C13—C14	−127.7 (3)	C31—C36—C35—C34	−0.8 (6)
C7—N2—C13—C14	55.0 (4)	C21—C22—C23—C24	0.0 (5)
C2—N2—C7—C8	−106.7 (4)	C12—C7—C8—C9	0.9 (5)
C13—N2—C7—C8	70.7 (4)	N2—C7—C8—C9	−176.3 (3)
C2—N2—C7—C12	76.1 (4)	C10—C9—C8—C7	−0.5 (6)
C13—N2—C7—C12	−106.6 (4)	C7—C12—C11—C10	−0.1 (6)
C8—C7—C12—C11	−0.6 (5)	C26—C25—C30—C29	−0.4 (6)
N2—C7—C12—C11	176.6 (3)	N4—C25—C30—C29	176.4 (4)
C14—C13—C18—C17	−0.8 (5)	C8—C9—C10—C11	−0.2 (6)
N2—C13—C18—C17	176.1 (3)	C12—C11—C10—C9	0.5 (7)
C16—C17—C18—C13	0.2 (5)	C1—N1—C2—N2	180.0 (3)
C2—N1—C1—O1	1.4 (6)	C1—N1—C2—S1	0.3 (5)
C2—N1—C1—C3	−178.9 (3)	C7—N2—C2—N1	−175.0 (3)
C4—C3—C1—O1	162.4 (4)	C13—N2—C2—N1	7.8 (4)
O2—C3—C1—O1	−13.5 (4)	C7—N2—C2—S1	4.8 (4)
C4—C3—C1—N1	−17.3 (5)	C13—N2—C2—S1	−172.4 (2)
O2—C3—C1—N1	166.8 (3)	Ni1—S1—C2—N1	−1.0 (3)
C30—C25—C26—C27	0.5 (6)	Ni1—S1—C2—N2	179.3 (2)
N4—C25—C26—C27	−176.3 (3)	C31—C32—C33—C34	−0.1 (6)
C32—C31—C36—C35	0.2 (5)	C4—C5—C6—O2	1.6 (5)
N4—C31—C36—C35	−178.6 (3)	C3—O2—C6—C5	−1.4 (4)
C18—C13—C14—C15	0.6 (5)	C26—C27—C28—C29	0.4 (7)
N2—C13—C14—C15	−176.3 (3)	C22—C23—C24—O4	1.0 (5)
C16—C15—C14—C13	0.2 (5)	C21—O4—C24—C23	−1.6 (5)
C19—N3—C20—N4	175.3 (3)	C36—C35—C34—C33	1.0 (6)
C19—N3—C20—S2	−3.1 (5)	C32—C33—C34—C35	−0.6 (6)
C25—N4—C20—N3	176.0 (3)	C27—C28—C29—C30	−0.2 (7)
C31—N4—C20—N3	−1.7 (4)	C25—C30—C29—C28	0.3 (7)
